# Evaluation of therapeutic effects of FAK inhibition in murine models of atherosclerosis

**DOI:** 10.1186/s13104-019-4220-5

**Published:** 2019-04-02

**Authors:** Takeshi Yamaura, Tatsuhiko Kasaoka, Naoko Iijima, Masaaki Kimura, Shinji Hatakeyama

**Affiliations:** 1 Novartis Institutes for BioMedical Research, Novartis Pharma K.K, Tsukuba, Ibaraki Japan; 2 Novartis Pharma AG, Basel, Switzerland

**Keywords:** Atherosclerosis, Focal adhesion kinase, apoE KO, LDLr KO

## Abstract

**Objective:**

Therapeutic effects of focal adhesion kinase (FAK) inhibition using a small molecule inhibitor was evaluated in apolipoprotein E (apoE) knockout (KO) and low-density lipoprotein receptor (LDLr) KO mouse atherosclerosis models.

**Results:**

The prevention trial consisted of an 8-week treatment with an FAK inhibitor concurrent treatment with a high fat (HF)/high cholesterol (HC) diet. The intervention trial consisted of 6- and 8-week treatment after 6- and 8-week pre-loading, respectively, of a HF/HC diet in apoE KO and LDLr KO mice, respectively. The inhibitor was admixed with a HF/HC diet and mice were given free access to the admixture. The FAK inhibitor exhibited marked inhibition against the development of the atherosclerosis in both of prevention and intervention trials at a dose of 0.03% without showing any remarkable toxic properties in biochemical examinations. These results indicated that FAK inhibition might be a possible candidate for novel therapeutic targets against atherosclerosis.

**Electronic supplementary material:**

The online version of this article (10.1186/s13104-019-4220-5) contains supplementary material, which is available to authorized users.

## Introduction

Focal adhesion kinase (FAK) is a non-receptor cytoplasmic tyrosine kinase that regulates multiple cell functions [1]. Activation of integrins and the growth factor receptors result in FAK autophosphorylation at Y397 and the presentation of suitable binding sites for proteins containing either Src homology 2 domain or phosphotyrosine binding domains. Association of FAK with PI3-kinase is thought to be necessary for FAK signaling pathway because phosphorylation of FAK on Y397 is required for cell proliferation, apoptosis and migration [2]. It is well known that elevated expression levels of FAK have been detected in various tumor samples and are closely correlated with invasive potential [3]. FAK is considered to be involved in inflammatory pathways such as leukocyte rolling, attachment and motility as well as migration of smooth muscle cells and endothelial cells [4, 5], but the roles of FAK in the development of atherosclerosis still remain to be clarified. Here we report the effect of FAK inhibition on the atherosclerosis in the spontaneous model using apoE KO and LDLr KO mice.

## Main text

### Methods

The mutant male mice, apoE KO (B6.129P2-*Apoe*^*tm1Unc*^/J) and LDLr KO (B6.129S7-*Ldlr*^*tm1Her*^/J) at the age of 7 weeks, were purchased from Charles River Laboratories, and acclimated to the facility for 7 days. The mice were housed at 25 °C with a 12:12 h light–dark cycle, and food and water were provided ad libitum. The HF/HC diet D12108 containing 1.25% cholesterol and 20% fat was purchased from Research Diets. A selective small molecule FAK inhibitor Compound 12 (Additional file 1: Figure S1; a 2-phenylamino-pyrimidin-4-ylamino-2,3-dihydro-isoindol-1-one derivative) was administrated as food admixture at a concentration of 0.03%. Compound 12 has an IC_50_ of 6 nM on FAK and no activity on insulin receptor kinase, insulin-like growth factor 1 receptor kinase, cyclin-dependent kinase 1 and Src (Additional file 2: Table S1) as described by Kawahara et al. [6]).

To assess the therapeutic effect of FAK inhibition, the following three different designs were applied: prevention (1) and therapeutic intervention (2) in apoE KO mice, and therapeutic intervention (3) in LDLr KO mice. The prevention trial consisted of 8-week concurrent treatment with the HF/HC diet following 1 week of pre-loading of the HF/HC diet (Additional file 3: Figure S2A). The therapeutic intervention trial consisted of 6- and 8-week treatment after 6- and 8-week pre-loading of the HF/HC diet in apoE KO and LDLr KO mice, respectively (Additional file 3: Figure S2B). Body weight and food intake were measured once weekly. The mice were euthanized under terminal inhalation anesthesia with isoflurane followed by blood collection from abdominal vein.

Atherosclerotic lesion area was evaluated in the aorta by *en face* analysis (Additional file 4: Figure S3A) and in aortic root by cross-sectional analysis (Additional file 4: Figure S3B). The aorta was subjected to Sudan IV staining, and total area of the plaque lesion (TAOL) and area of whole aorta from arch without the region of brachiocephalic artery to common iliac artery (AOWA) were measured using the Definiens imaging software. Area of lesion (%) was calculated by the following formula; Area of lesion (%) = TAOL/AOWA × 100. After en face analysis, the aorta was washed and then blotted dry, weighed, minced, and total lipid was extracted with chloroform/methanol. Total and free cholesterol levels in the aortic extracts were determined with an enzymatic colorimetric assay (Wako Pure Chemical Industries Ltd.). Cross sections were cut at 5 μm thickness and 6 serial sections were collected every 50 μm along the aortic root. Lesion area analysis was made on the cross sections subjected to hematoxylin and eosin (HE) staining. Three representative sections derived from 3 segments were quantified per mouse. Lesion area values from the 3 sections were summed up. Histological features using serial cross sections were subjected to staining for EvG and anti Mac-3 antigen. Clinical biochemistry markers in serum was evaluated using DRICHEM 7000 (FUJIFILM Medical, Japan). Statistical comparison of control and compound 12-treated groups was carried out with a Mann–Whitney test (two-tailed) using GraphPad Prism.

### Results and discussion

**Fig. 1:**
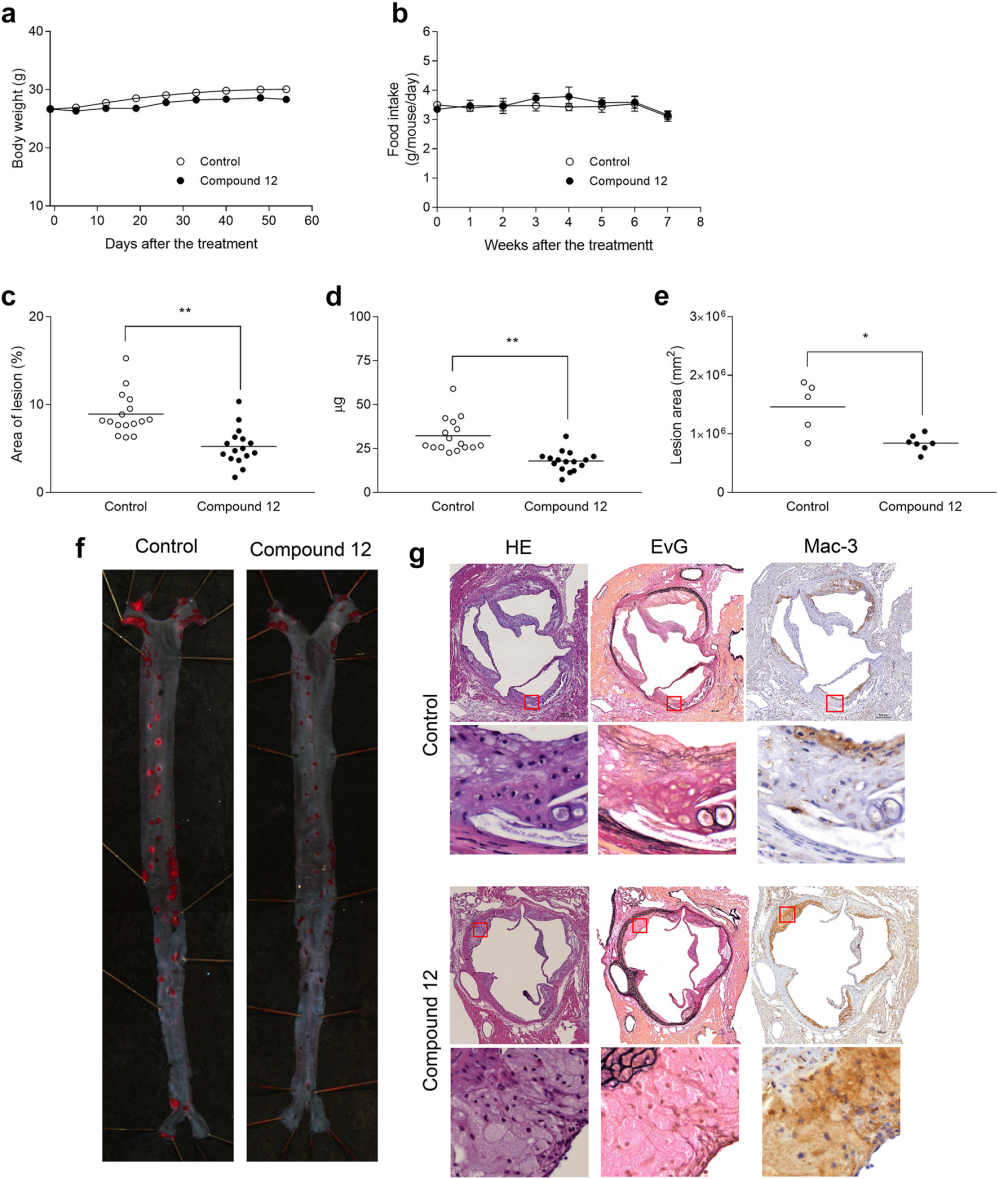
Prevention in apoE KO mice. Values are expressed as mean ± SEM (**a**, **b**) or mean with scatter plots (**c**–**e**) (n = 16). (A) Body weight change. **b** Food intake. **c** Lesion area in en face analysis. **P < 0.01 as compared with control. **d** Cholesterol content in aorta. **P < 0.01 as compared with control. **e** Lesion area of aortic root (n = 5–7). *P < 0.05 as compared with control. **f** Aorta with Sudan IV staining. **g** Histological features using serial cross sections subjected to HE, EvG and anti Mac-3 antigen. In the EvG staining, elastic fibers and nuclei appear in dark purple, collagen fibers in red, smooth muscle and cytosol between internal and external laminae in yellow

**Fig. 2:**
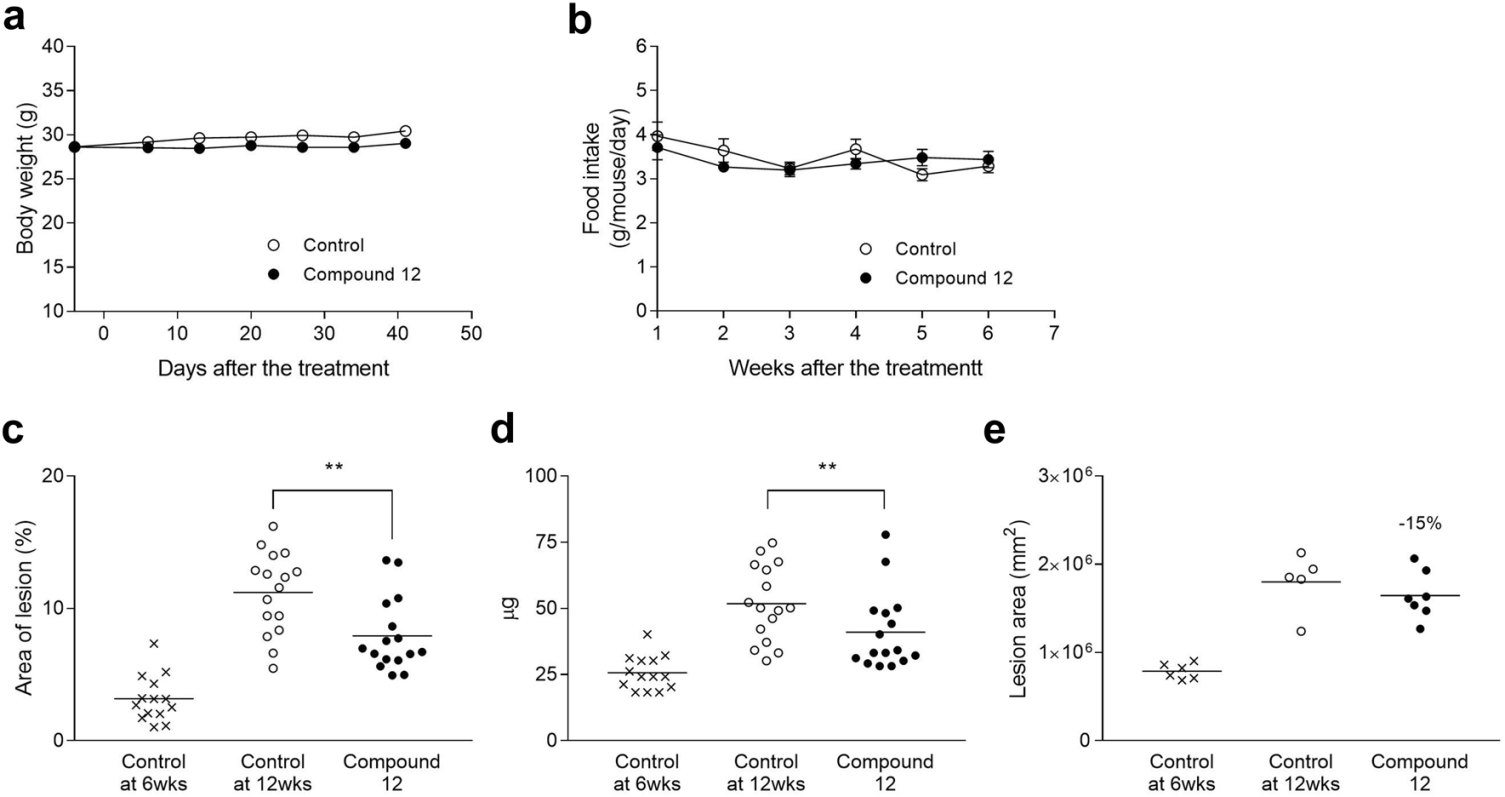
Therapeutic intervention in apoE KO mice. Values are expressed as mean ± SEM (**a**, **b**) or mean with scatter plots (**c**–**e**) (n = 16). **a** Body weight change. **b** Food intake. **c** Lesion area in en face analysis. **; *P* < 0.01 as compared with control. **d** Cholesterol content in aorta. *P < 0.05 as compared with control. **e** Lesion area of aortic root (n = 5–7). *P < 0.05 as compared with control

**Fig. 3:**
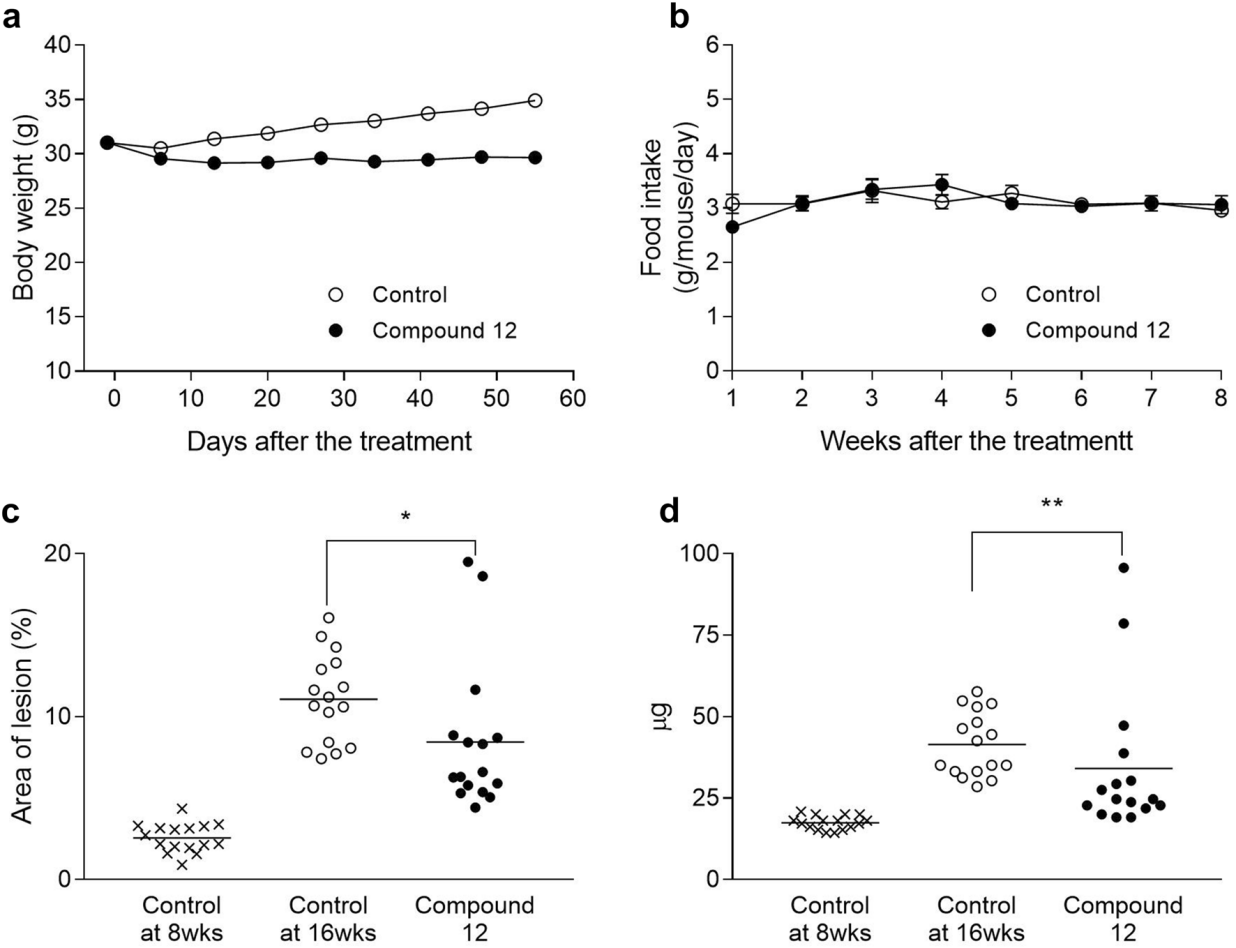
Therapeutic intervention in LDLr KO mice. Values are expressed as mean ± SEM (**a**, **b**) or mean with scatter plots (**c**, **d**) (n = 16). (A) Body weight change. **b** Food intake. **c** Lesion area in *en face* analysis. *P < 0.05 as compared with control. **d** Cholesterol content in aorta. **P < 0.01 as compared with control

The effect of a selective FAK inhibitor, Compound 12 [6] was evaluated in apoE KO and LDLr KO mouse spontaneous models of atherosclerosis in 2 different designs, prevention and therapeutic intervention trials. Compound 12 was generally well tolerated and there were no remarkable changes in body weight and food intake in 3 trials: prevention in apoE KO mice (Fig. 1a, b), therapeutic intervention in apoE KO mice (Fig. 2a, b) and in LDLr KO mice (Fig. 3a, b).

Compound 12 showed a marked inhibitory effect on the development of the atherosclerosis in the prevention trial using apoE KO mice and the intervention trials using apoE KO and LDLr KO mice. In the prevention trial in apoE KO mice, Compound 12 significantly reduced the lesion area of the aorta by 41.2% (Fig. 1c; 8.92% in Control and 5.24% in Compound 12), and also significantly reduced the cholesterol content (Fig. 1d) and lesion area of aortic root (Fig. 1e). A representative picture of en face analysis of the aorta stained withF Sudan IV is presented in Fig. 1f. Histological features using serial cross sections with HE, EvG and anti Mac-3 are presented in Fig. 1g (higher magnifications in lower panels). Interestingly, in atherosclerotic plaques from the control group, cholesterol crystals, fibrous and lipid core regions were observed displaying histological features similar to those seen in human, while in atherosclerotic plaques from the Compound 12-treated group, foam cell rich, reduced fibrous and lipid core regions were observed.

In the therapeutic intervention trials in apoE KO and LDLr KO mice, Compound 12 significantly reduced the lesion area of the aorta by 40.9% in apoE KO mice (Fig. 2c; 8.03% in Control at 12 weeks and 4.75% in Compound 12 normalized to Control at 6 weeks) and by 30.9% in LDLr KO mice (Fig. 3c; 8.51% in Control at 16 weeks and 5.83% in Compound 12 normalized to Control at 8 weeks), and also significantly reduced the cholesterol content (Figs. 2d, 3d). The lesion area of aortic root appeared to be reduced in apoE KO mice (Fig. 2e).

There were some changes in clinical biochemistry markers (Additional file 5: Table S2): a significant or a trend of reduction in ALT, LDH and T-Chol may suggest a protective effect of FAK inhibition on organs under the HF/HC condition, and in contrary, a significant or a trend of increase in ALP, TG and CRE could indicate on-target safety flags. The mechanism behind T-Chol lowering is unclear.

The precise mechanism of how FAK inhibition could prevent or inhibit atherosclerosis remains unclear although a pathological role of FAK in atherosclerosis has been reported, such as its activation by remnant lipoproteins through RhoA and s1-integrin [7], by angiotensin II through Akt-mTOR-NF-κB signaling pathway [8], and by oxidized LDL through p90 ribosomal S6 kinase family proteins [9]. These results would suggest that FAK might play an important role in the development of atherosclerosis and FAK inhibition might be a possible candidate for novel therapeutic targets for the treatment of atherosclerosis.

## Limitations

In the experiments described in this report, we only evaluated the efficacy of the FAK inhibitor and did not assess any biomarkers related to FAK activation or inhibition, and atherosclerosis. Further assessment in various pharmacodynamic and functional assays will be required to elucidate the mechanism of action of FAK inhibition as well as to reveal a pathological role of FAK in atherosclerosis.

## Additional files

**Additional file 1: Figure S1.** The structure of Compound 12. Chemical name: 7-[5-Chloro-2-(2,4-dimethoxy-phenylamino)-pyrimidin-4-ylamino]-2-methyl-4-[4-(4-methyl-piperazin-1-yl)-piperidin-1-yl]-2,3-dihydro-isoindol-1-one.

**Additional file 2: Table S1.** IC_50_ values; Kinase inhibition selectivity of compound 12.

**Additional file 3: Figure S2.** Overview of efficacy trials. (A) Prevention in apoE KO mice, (B) therapeutic intervention in apoE KO mice (6 + 6 weeks) and LDLr KO mice (8 + 8 weeks).

**Additional file 4: Figure S3.** Atherosclerotic lesion area analysis. Atherosclerotic lesion area was evaluated in the aorta by *en face* analysis (A) and in aortic root by cross-sectional analysis (B).

**Additional file 5: Table S2.** Clinical biochemistry markers; Data expressed as mean ± SEM.

## Abbreviations

ALB: albumin
ALP: alkaline phosphatase
ALT: alanine aminotransferase
AOWA: area of whole aorta
apoE: apolipoprotein E
AST: aspartate aminotransferase
ATP: adenosine triphosphate
BUN: blood urea nitrogen
CPK: creatine phosphokinase
CRE: creatinine
EvG: Elastica van Gieson
FAK: focal adhesion kinase
Glc: glucose
HC: high cholesterol
HE: hematoxylin eosin
HF: high fat
LDH: lactate dehydrogenase
LDLr: low-density lipoprotein receptor
SMA: smooth muscle actin
TAOL: total area of the plaque lesion
T-BIL: total bilirubin
T-Chol: total cholesterol
TG: triglyceride
