# Supplementary material for: Evaluation of therapeutic effects of FAK inhibition in murine models of atherosclerosis
Source: BMC Res Notes. 2019 Apr 2;12:200. doi: 10.1186/s13104-019-4220-5 (PMC6446301; doi:10.1186/s13104-019-4220-5)
Supplement: Supplementary file 1 — Additional file 1: Figure S1. The structure of Compound 12. Chemical name: 7-[5-Chloro-2-(2,4-dimethoxy-phenylamino)-pyrimidin-4-ylamino]-2-methyl-4-[4-(4-methyl-piperazin-1-yl)-piperidin-1-yl]-2,3-dihydro-isoindol-1-one. [file 13104_2019_4220_MOESM1_ESM.docx]

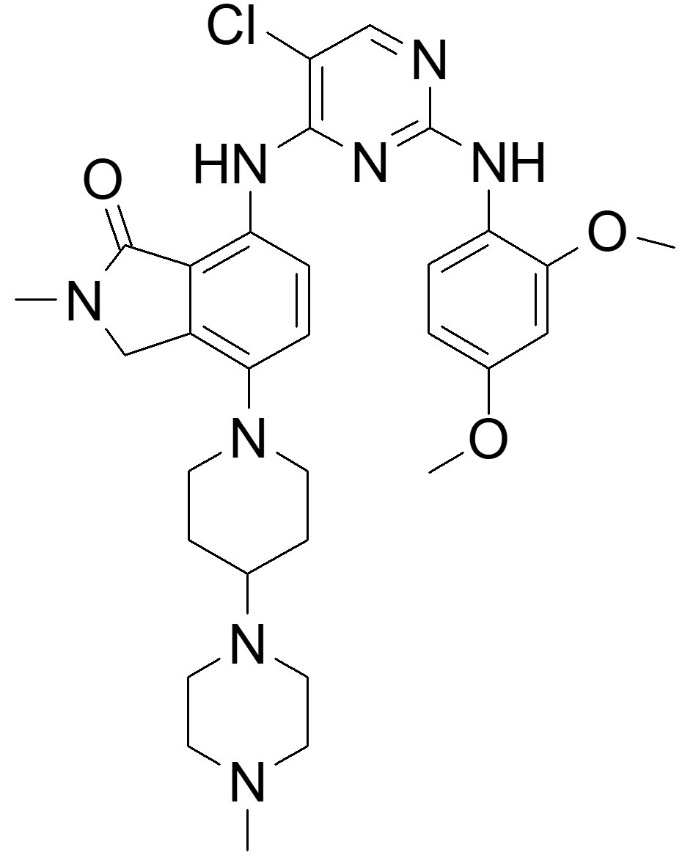


**The structure of Compound 12**

Chemical name: 7-[5-Chloro-2-(2,4-dimethoxy-phenylamino)-pyrimidin-4-ylamino]-2-methyl-4-[4-(4-methyl-piperazin-1-yl)-piperidin-1-yl]-2,3-dihydro-isoindol-1-one.
