# Supplementary material for: Evaluation of therapeutic effects of FAK inhibition in murine models of atherosclerosis
Source: BMC Res Notes. 2019 Apr 2;12:200. doi: 10.1186/s13104-019-4220-5 (PMC6446301; doi:10.1186/s13104-019-4220-5)
Supplement: Supplementary file 2 — Additional file 2: Table S1. IC50 values; Kinase inhibition selectivity of compound 12. [file 13104_2019_4220_MOESM2_ESM.docx]

**Table S1: IC_50_ values**

| Enzyme | IC_50_ (μM) |
| --- | --- |
| FAK | 0.006 |
| IRK | >3.3 |
| IGF-1R | >10 |
| CDK1 | >10 |
| c-Src | >10 |

Values are expressed as means. Kinase activity was evaluated by using radiolabeled ATP, DELFIA^®^ or TR-FRET method [6].
