# Supplementary material for: Evaluation of therapeutic effects of FAK inhibition in murine models of atherosclerosis
Source: BMC Res Notes. 2019 Apr 2;12:200. doi: 10.1186/s13104-019-4220-5 (PMC6446301; doi:10.1186/s13104-019-4220-5)
Supplement: Supplementary file 3 — Additional file 3: Figure S2. Overview of efficacy trials. (A) Prevention in apoE KO mice, (B) therapeutic intervention in apoE KO mice (6 + 6 weeks) and LDLr KO mice (8 + 8 weeks). [file 13104_2019_4220_MOESM3_ESM.docx]

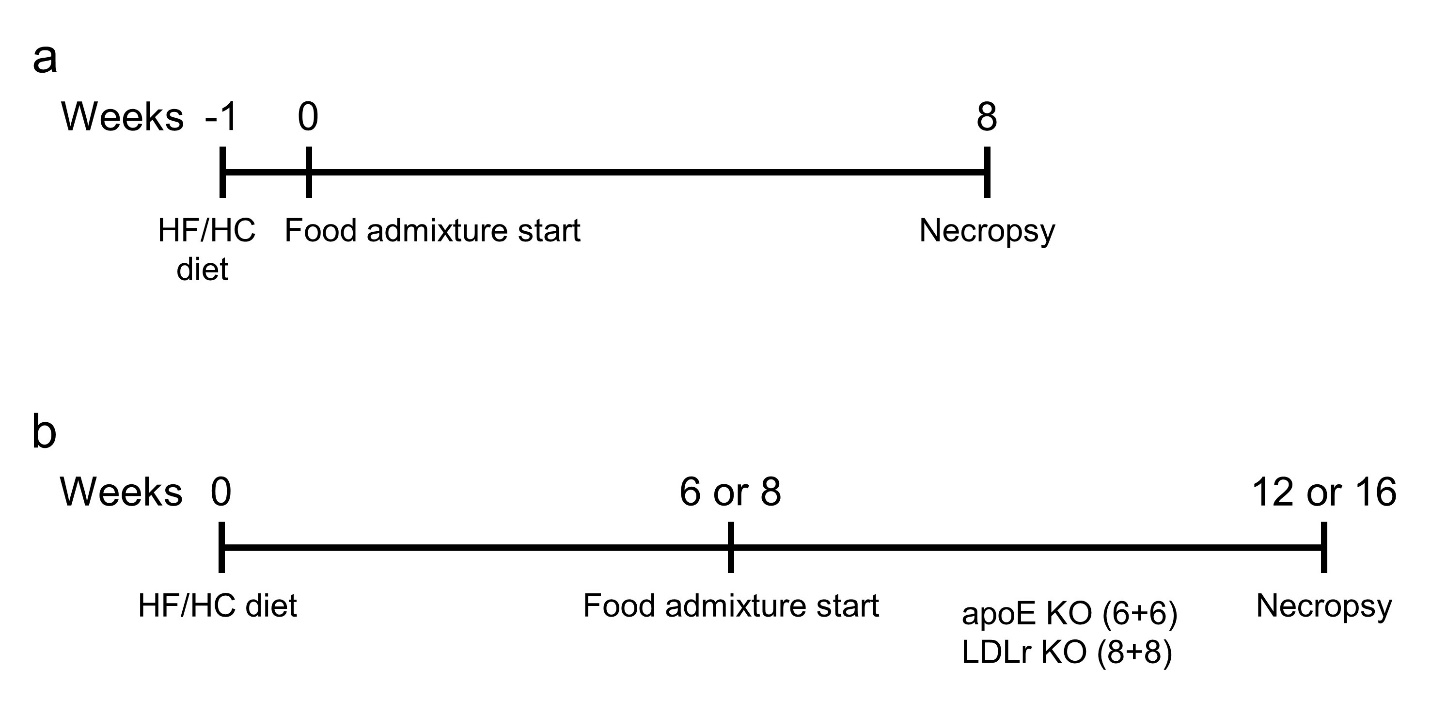


**Overview of efficacy trials**

(A) Prevention in apoE KO mice, (B) therapeutic intervention in apoE KO mice (6 + 6 weeks) and LDLr KO mice (8 + 8 weeks)
