# Supplementary material for: Evaluation of therapeutic effects of FAK inhibition in murine models of atherosclerosis
Source: BMC Res Notes. 2019 Apr 2;12:200. doi: 10.1186/s13104-019-4220-5 (PMC6446301; doi:10.1186/s13104-019-4220-5)
Supplement: Supplementary file 4 — Additional file 4: Figure S3. Atherosclerotic lesion area analysis. Atherosclerotic lesion area was evaluated in the aorta by en face analysis (A) and in aortic root by cross-sectional analysis (B). [file 13104_2019_4220_MOESM4_ESM.docx]

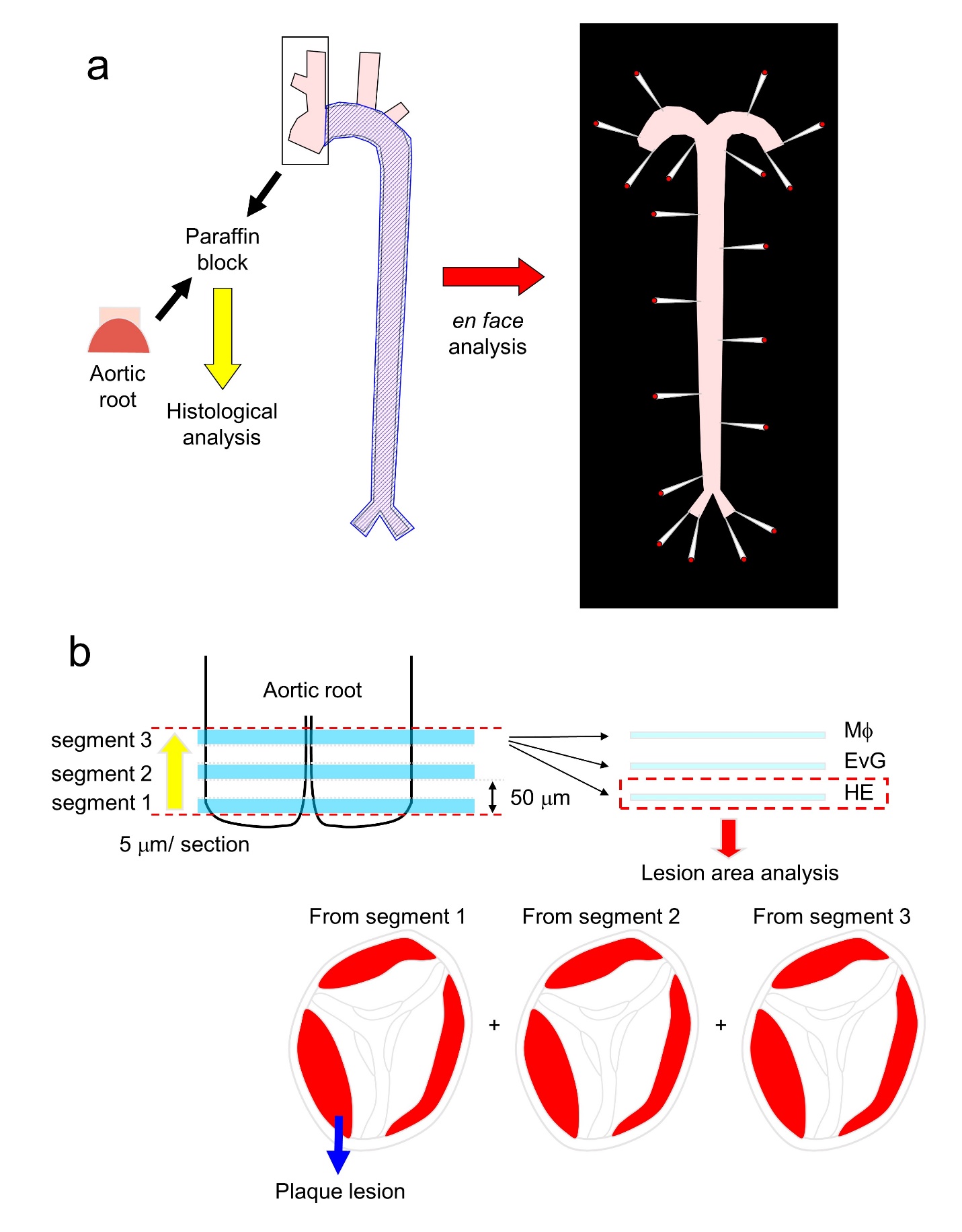


**Atherosclerotic lesion area analysis**

Atherosclerotic lesion area was evaluated in the aorta by *en face* analysis (A) and in aortic root by cross-sectional analysis (B).
