# Supplementary material for: Evaluation of therapeutic effects of FAK inhibition in murine models of atherosclerosis
Source: BMC Res Notes. 2019 Apr 2;12:200. doi: 10.1186/s13104-019-4220-5 (PMC6446301; doi:10.1186/s13104-019-4220-5)
Supplement: Supplementary file 5 — Additional file 5: Table S2. Clinical biochemistry markers; Data expressed as mean ± SEM. [file 13104_2019_4220_MOESM5_ESM.docx]

**Table S2: Clinical biochemistry markers**

| Model | apoE KO | | | | LDLr KO | |
| --- | --- | --- | --- | --- | --- | --- |
| Trial | Prevention (8 weeks) | | Intervention (6 + 6 weeks) | | Intervention (8 + 8 weeks) | |
| Group | Control | Compound 12 | Control | Compound 12 | Control | Compound 12 |
| ALB (g/dL) | 2.4 ± 0.04 | 2.2 ± 0.07^*^ | 2.3 ± 0.03 | 2.3 ± 0.04 | 2.3 ± 0.03 | 2.2 ± 0.05 |
| AST (U/L) | 126 ± 19 | 115 ± 15 | 101 ± 11 | 108 ± 7.0 | 89 ± 11 | 97 ± 8.0 |
| ALT (U/L) | 40 ± 7.4 | 25 ± 1.6 | 29 ± 3.4 | 26 ± 1.3 | 38 ± 3.1 | 24 ± 1.6^**^ |
| LDH (U/L) | 1,012 ± 143 | 535 ± 47^**^ | 743 ± 53 | 590 ± 36^*^ | 383 ± 34 | 295 ± 38 |
| ALP (U/L) | 446 ± 48 | 554 ± 31^**^ | 380 ± 56 | 469 ± 37^***^ | 249 ± 40 | 406 ± 34^**^ |
| CPK (U/L) | 136 ± 15 | 700 ± 362 | 168 ± 41 | 227 ± 48 | 70 ± 9.6 | 196 ± 76^**^ |
| T-BIL (mg/dL) | 0.5 ± 0.05 | 0.5 ± 0.03 | 0.5 ± 0.06 | 0.5 ± 0.05 | 0.5 ± 0.05 | 0.6 ± 0.05 |
| T-Chol (mg/dL) | 1,503 ± 71 | 1,221 ± 102^*^ | 1,197 ± 44 | 1,016 ± 53^*^ | 1,150 ± 49 | 714 ± 51^***^ |
| TG (mg/dL) | 72 ± 5.7 | 94 ± 8.4^*^ | 68 ± 3.7 | 93 ± 6.7^**^ | 294 ± 19 | 273 ± 21 |
| BUN (mg/dL) | 37 ± 1.3 | 41 ± 2.5 | 41 ± 2.5 | 42 ± 2.5 | 31 ± 0.92 | 32 ± 1.2 |
| CRE (mg/dL) | 0.2 ± 0.01 | 0.3 ± 0.02^***^ | 0.2 ± 0.02 | 0.3 ± 0.02^***^ | 0.2 ± 0.01 | 0.3 ± 0.02^***^ |
| Insulin (pg/mL) | 576 ± 88 | 751 ± 109 | - | - | - | - |
| Glc (mg/dL) | 164 ± 24 | 167 ± 16 | - | - | - | - |

Data are mean ± SEM. *; *P* < 0.05, **; *P* < 0.01 ***; *P* < 0.001 as compared with the control.
